# Gene expression in immortalized versus primary isolated cardiac endothelial cells

**DOI:** 10.1038/s41598-020-59213-x

**Published:** 2020-02-10

**Authors:** Lisa Deng, Luisa Pollmeier, Qian Zhou, Stella Bergemann, Christoph Bode, Lutz Hein, Achim Lother

**Affiliations:** 1 Institute of Experimental and Clinical Pharmacology and Toxicology, Faculty of Medicine, University of Freiburg, Freiburg im Breisgau, Germany; 2 Heart Center Freiburg University, Department of Cardiology and Angiology I, Faculty of Medicine, University of Freiburg, Freiburg im Breisgau, Germany; 3 BIOSS Centre for Biological Signaling Studies, University of Freiburg, Freiburg im Breisgau, Germany

**Keywords:** Angiogenesis, Cardiovascular genetics

## Abstract

Endothelial cells take pivotal roles in the heart and the vascular system and their differentiation, subspecification and function is determined by gene expression. A stable, *in vitro* cardiac endothelial cell line could provide high cell numbers as needed for many epigenetic analyses and facilitate the understanding of molecular mechanisms involved in endothelial cell biology. To test their suitability for transcriptomic or epigenetic studies, we compared the transcriptome of cultured immortalized mouse cardiac endothelial cells (MCEC) to primary cardiac endothelial cells (pEC). Whole transcriptome comparison of MCEC and pEC showed a correlation of 0.75–0.77. Interestingly, correlation of gene expression declined in endothelial cell-typical genes. In MCEC, we found a broad downregulation of genes that are highly expressed in pEC, including well-described markers of endothelial cell differentiation. Accordingly, systematic analysis revealed a downregulation of genes associated with typical endothelial cell functions in MCEC, while genes related to mitotic cell cycle were upregulated when compared to pEC. In conclusion, the findings from this study suggest that primary cardiac endothelial cells should preferably be used for genome-wide transcriptome or epigenome studies. The suitability of *in vitro* cell lines for experiments investigating single genes or signaling pathways should be carefully validated before use.

## Introduction

Endothelial cells are the most abundant non-myocyte cell type in the adult heart^1^. They form the inner layer of blood vessels and the capillary network that provide oxygen and nutrition supply to the heart tissue. However, cardiac endothelial cell function is by far more complex. Endothelial cells modulate coronary blood flow by secretion of vasoconstrictive and vasodilative factors^2^. Via cytokines and chemokines endothelial cells interact with immune cells and control vascular permeability, leukocyte adhesion and transmigration^3^. Of note, endothelial cells show organotypic heterogeneity with highly specific functions, e.g. formation of the blood-brain-barrier or the renal glomerular filtration barrier, which is reflected by their transcriptome and translatome^4–7^. Cardiac endothelial cells take specific functions in energy metabolism and substrate supply^8^. In addition, paracrine signaling from endothelial cells may promote cardiac myocyte growth and fibroblast activity^2,3^. Endothelial cell differentiation and functional subspecification is determined by gene expression under tight control of transcription factors and epigenetic modifiers^5,9,10^. Thus, analyzing gene expression is a promising approach to understand the molecular mechanisms involved in these processes. Mouse models are widely used for *in vivo* studies, however, isolation of primary endothelial cells from mouse hearts is time-consuming, variable, and provides limited cell numbers. A stable, *in vitro* mouse cardiac endothelial cell line could provide high cell numbers as needed for many epigenetic analyses, facilitate functional assays using pharmacological, genetic or epigenetic interventions *in vitro*, and help to reduce laboratory animal use.

In order to provide high cell numbers, mouse cardiac endothelial cells (MCEC) immortalized by transfection with lentiviral vectors carrying SV40 T-cell antigen and human telomerase have been created for *in vitro* use^11,12^. The aim of the present study was to assess the transcriptome of immortalized mouse cardiac endothelial cells in comparison to primary cardiac endothelial cells and thereby to test their suitability for transcriptomic or epigenetic studies.

## Results

### The transcriptome of immortalized versus primary isolated endothelial cells shows low correlation

**Figure 1:**
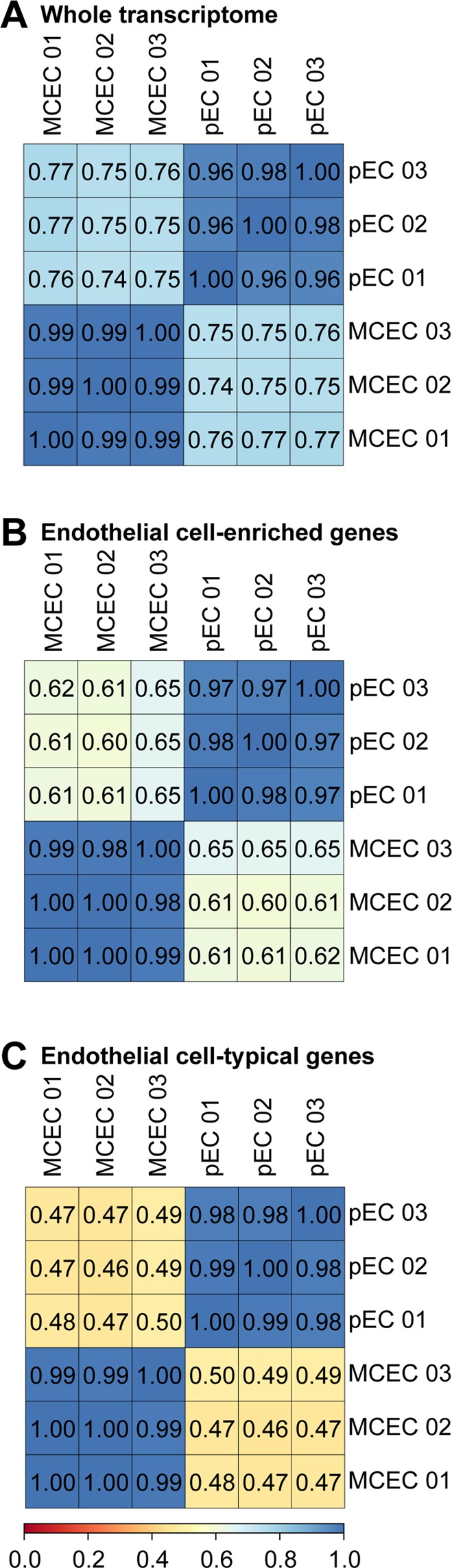
Correlation of immortalized versus primary cardiac endothelial cell gene expression. Gene expression in immortalized mouse cardiac endothelial cells (MCEC) and primary isolated mouse cardiac endothelial cells (pEC). Spearman’s correlation was calculated for all ENSEMBL annotated genes (**A**), for genes enriched in pECs (**B**) and for pEC-typical genes (**C**).

We performed RNAseq from cultured immortalized mouse cardiac endothelial cells (MCEC) and compared them to gene expression data from primary isolated mouse cardiac endothelial cells (pEC)^6^. Within their group, biological replicates showed a high correlation of ≥0.99 for MCEC and ≥0.96 for pEC of gene expression of all ENSEMBL annotated genes (>1 FPKM in pEC or MCEC, Fig. 1A). In contrast, comparison of the different groups revealed a correlation of 0.75–0.77 between MCEC and pEC (Fig. 1A). Based on our previous findings^6^, we restricted the analysis to endothelial cell-enriched genes (log_2_ fold-change pEC versus heart tissue >0) or endothelial cell-typical genes (log_2_ fold-change pEC versus heart tissue >3) to avoid any bias due to contamination of the pEC by non-endothelial cell RNA. Interestingly, correlation of gene expression in MCEC and pEC was lower in endothelial cell-enriched and further declined in endothelial cell-typical genes (Fig. 1B,C) indicating differential expression of genes that are highly expressed in endothelial cells.

### Endothelial cell-typical genes are downregulated in MCEC

**Figure 2:**
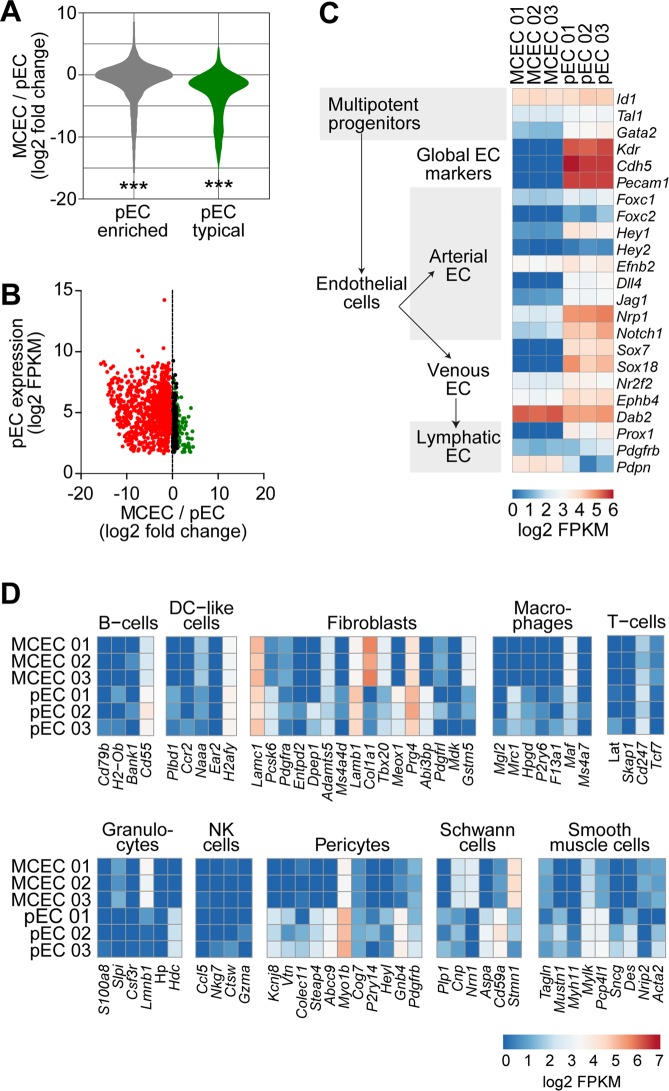
Differential gene expression of immortalized versus primary cardiac endothelial cells. Log_2_ ratio of gene expression in immortalized (MCEC) versus primary endothelial cell (pEC)-enriched or -typical genes was tested versus a theoretical mean of 0 (**A**), ***P < 0.001, one-sample t-test) and correlated with gene expression in pEC (**B**). Expression of marker genes for endothelial cells (**C**) or other cardiac cell types (**D**) was determined in MCEC and pEC. n = 3 per group. FPKM, fragments per kilobase of transcript per million fragments mapped.

In total, we identified 6,873 endothelial cell-enriched genes differentially expressed in MCEC versus pEC (2,799 genes up, 4,074 genes down; q < 0.05). Mean fold-change of endothelial cell-enriched genes in MCEC versus pEC was negative, indicating a broad downregulation of endothelial cell genes in MCEC (Fig. 2A). This effect was even aggravated for endothelial cell-typical genes (Fig. 2A). Apparently, a number of genes that are highly expressed in pEC were downregulated in MCEC (Fig. 2B). These included well-described markers of endothelial cell differentiation^6,13^ such as cadherin 5 (*Cdh5*) or platelet endothelial cell adhesion molecule 1 (*Pecam1, CD31*) (Fig. 2C). The use of different cell culture media did not affect marker gene expression (Supplementary Figure 1). We assessed the expression of non-endothelial cell marker genes^14^ in MCEC and pEC. However, neither MCEC nor pEC expressed genes assignable to any other cardiac cell type tested (Fig. 2D).

### Expression of genes associated with endothelial cell function is altered in MCEC

**Figure 3:**
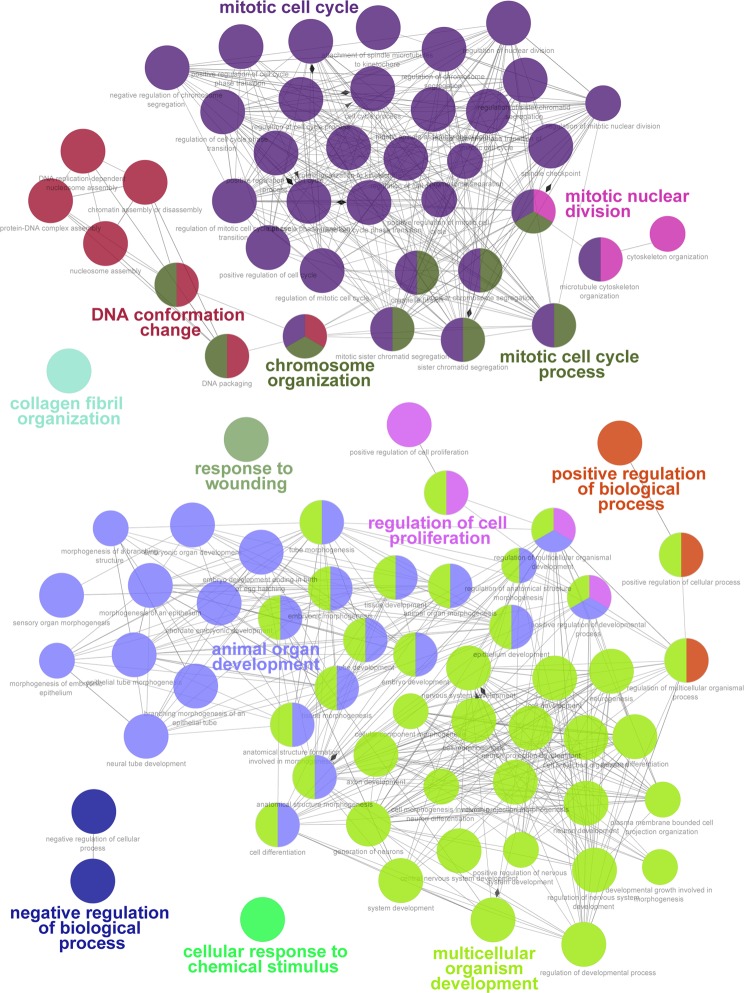
Molecular pathway analysis of genes upregulated in MCEC. Enrichment (P < 0.01) of biological processes derived from Gene Ontology (GO) among the 1000 genes that were most significantly upregulated in immortalized versus primary endothelial cells (q < 0.05) was analyzed using ClueGO. n = 3 per group.

**Figure 4:**
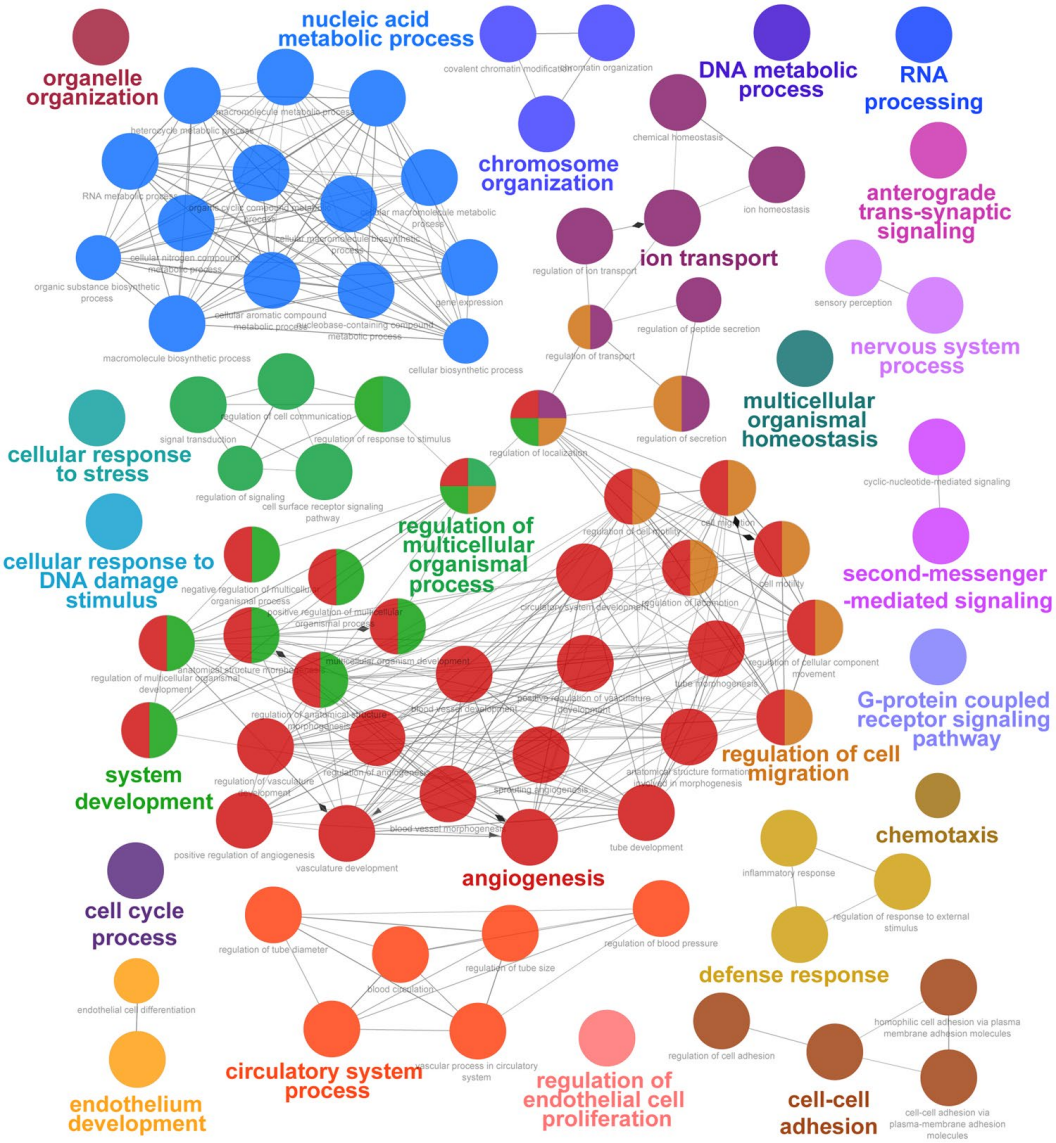
Molecular pathway analysis of genes downregulated in MCEC. Enrichment (P < 0.01) of biological processes derived from Gene Ontology (GO) among the 1000 genes that were most significantly downregulated in immortalized versus primary endothelial cells (q < 0.05) was analyzed using ClueGO. n = 3 per group.

To systematically evaluate the distinct biological role of genes showing differential expression in MCEC versus pEC, we analyzed their association with biological processes using the gene ontology database^15^. In MCEC, we found genes upregulated that were associated with mitotic cell cycle, DNA conformation or chromatin, and organ development (Fig. 3). In contrast, genes that were associated with typical endothelial cell functions such as angiogenesis, circulatory system processes or endothelium development were downregulated in MCEC (Fig. 4).

### Immortalization affects the expression of angiogenesis-related genes

**Figure 5:**
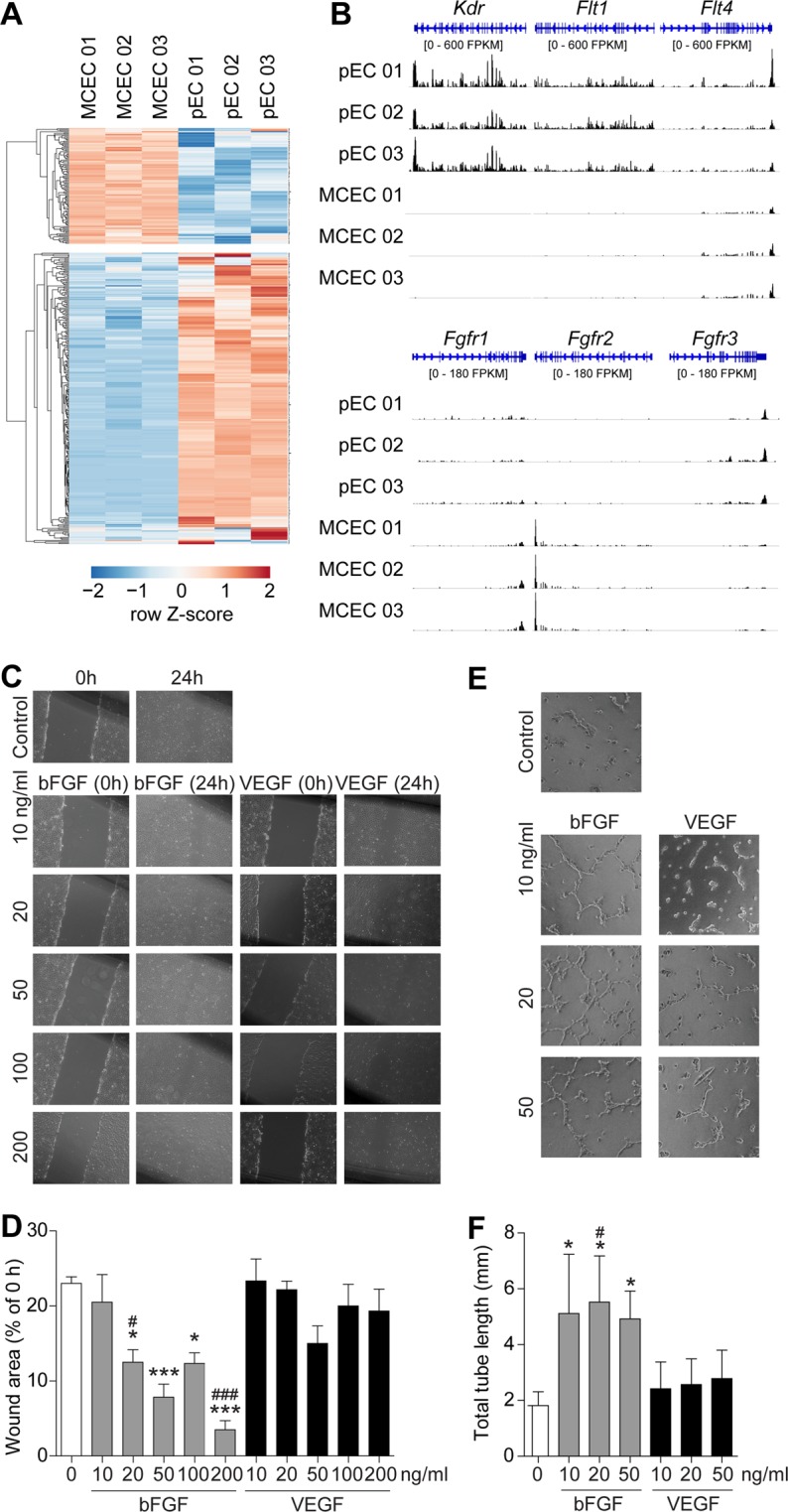
Angiogenesis-related gene expression in MCEC. The subset of genes related to gene ontology terms GO:0001525 (angiogenesis), GO:0003013 (circulatory system process), GO:0003158 (endothelium development) or GO:0045446 (endothelial cell differentiation) among genes that were up- or downregulated (n = 3 per group, q < 0.05) in immortalized (MCEC) versus primary endothelial cells (pEC) (**A**). Representative traces showing gene expression of receptors for vascular endothelial cell growth factor (*Kdr, Flt1, Flt4*) or fibroblast growth factor (*Fgfr1, Fgfr2, Fgfr3*) (**B**). Functional capacity of MCECs in response to VEGF and bFGF was assessed in scratch assays (**C**,**D**, n = 6 per group) and tube formation assays (**E**,**F**, n = 3 per group). One-way ANOVA with Newman-Keuls multiple comparison test. *P < 0.05; **P < 0.01; ***P < 0.001 vs. unstimulated control, ^#^P < 0.05; ^###^P < 0.001 vs. concentration-equivalent VEGF. FPKM, fragments per kilobase of transcript per million fragments mapped.

We focused on genes associated with angiogenesis, circulatory system processes or endothelium development and identified in total 387 genes being differentially regulated in MCEC versus pEC (110 genes up, 277 genes down; q < 0.05, Fig. 5A). Interestingly, we observed marked differences in the expression of receptors for vascular endothelial cell growth factor or fibroblast growth factor between MCEC and pEC (Fig. 5B). To evaluate the impact of these findings on endothelial cell-typical functional capacities of MCECs, i.e. migration and sprouting toward VEGF and bFGF, we performed scratch assays and tube formation assays. As demonstrated in the scratch assay, stimulation with bFGF strongly enhanced cell migration towards the scratch area compared to control treatment (Fig. 5C,D). In contrast, stimulation with VEGF had no significant effect on cell migration. Likewise, bFGF markedly increased total sprout length in the tube formation assay, while no response to VEGF was detectable (Fig. 5E,F).

## Discussion

The key finding from this study is that *in vitro* cultivated immortalized mouse cardiac endothelial cells showed a disturbed transcriptome that is largely different from primary isolated cardiac endothelial cells. Of note, the differences between MCEC and pEC were pronounced in endothelial cell-typical genes including marker genes such as cadherin 5 (*Cdh5*) or platelet endothelial cell adhesion molecule 1 (*Pecam1*).

MCEC are a frequently used *in vitro* cell system^11,12,16–19^. Though they showed endothelial cell-like behavior in *in vitro* assays, MCEC apparently lost key endothelial cell characteristics in their gene expression program. We hypothesized that MCEC might have transdifferentiated into another cell type. However, when assessing the expression of typical marker genes for other cardiac cell types derived from single cell RNA sequencing studies we could not identify a characteristic pattern. In contrast, MCEC showed an upregulation of genes associated with mitosis or DNA conformation, likely reflecting their rapid proliferation rate. Similar findings have been reported for other immortalized endothelial and non-endothelial cell lines. Immortalization of pterygeum fibroblasts^20^, foreskin fibroblasts^21^ or breast epithelial cells^22^ lead to an upregulation of cell cycle genes or oncogenes. Most strikingly, comparison of human umbilical vein endothelial cells (HUVEC) and EA.hy926, an immortalized HUVEC line, revealed an upregulation of genes related to cell cycle control and apoptosis in immortalized endothelial cells^23^. In line with our findings, decreased expression of endothelial cell marker genes has been reported earlier for other immortalized endothelial cells lines^24^.

Recently, we and others have analyzed the transcriptome of primary cardiac endothelial cells^6,25,26^. The main findings of these studies are that the cardiac endothelial cell transcriptome is specific versus other cardiac cell types^6^, specific versus endothelial cells from other organs^6^ and dynamically changing during development or disease^25,26^. Thus, it would be an asset to use cardiac endothelial cells and not endothelial cells from other sources to properly assess distinct signaling pathways and to improve the validity of *in vitro* experiments.

In conclusion, the findings from this study suggest that primary cardiac endothelial cells should preferably be used for genome-wide transcriptome or epigenome studies. The suitability of *in vitro* cell lines for experiments investigating single genes or signaling pathways should be carefully validated before use.

## Methods

### Cell culture

Immortalized mouse cardiac endothelial cells (MCEC) were obtained from Cedarlane (Burlington, Ontario, Canada) at passage 38. Unless otherwise stated cells were cultured in DMEM (Gibco) without pyruvate containing 10 mM Penicillin/Streptomycin (Gibco), 10 mmol/L HEPES (Carl Roth) and 5% FCS (Merck Millipore) according to the manufacturer’s instructions and used at passage 40.

### *In vitro* scratch assay

*In vitro* scratch assay was performed as described^27^. Briefly, MCEC at passage 42–53 were seeded into 6 cm dishes and grown to monolayer. A scratch was scraped into the monolayer using a pipet tip. Cell debris were removed and cells were stimulated in culture medium with VEGF (10, 20, 50, 100 or 200 ng/ml) or bFGF (10, 20, 50, 100 or 200 ng/ml) for 24 hours to promote migration. Images were taken and the remaining wound area (% of 0 hours) was measured with Zeiss Axioplan 2 using Axiovision Rel 4.8. Cells kept in culture medium were used as unstimulated control.

### Tube formation assay

Culture plates were coated with Matrigel (BD Bioscience) according to the manufacturer’s instructions. A total 2.5 × 10^4^ MCEC were cultured on Matrigel in 1% MCEC medium and stimulated with vehicle, VEGF (10, 20 or 50 ng/ml) or bFGF (10, 20 or 50 ng/ml) for 8 hours at 37 °C. Cells were fixed with 4% PFA and pictures were taken from four random microscopic fields.

### RNA preparation and RNA sequencing

For RNAseq experiments, cultured endothelial cells were washed twice with PBS and total RNA was isolated using an AllPrep DNA/RNA Micro Kit (Qiagen). RNA quality was assessed using a Bioanalyzer (2100 Bioanalyzer, Agilent). cDNA amplification was performed using the Ovation RNA-Seq System V2 Kit (NuGEN) and cDNA was fragmented to approximately 350 bp fragments using a sonication device (Bioruptor, Diagenode). 100 ng cDNA were used for library construction with the NEB Next Ultra DNA Library Prep Kit for Illumina (New England BioLabs) and amplified by fluorescence-controlled PCR. Size selection was performed with AMPure XP Beads (Beckmann Coulter) and verified in a Bioanalyzer. Libraries were sequenced on a HiSeq. 2500 deep sequencing unit (50 bp, paired-end, Illumina) at the Max Planck Institute of Immunobiology and Epigenetics, Freiburg. RNAseq data are available at the NCBI Gene Expression Omnibus repository (BioProject ID PRJNA524803).

### Primary cardiac endothelial cells

We re-analyzed RNAseq data from primary mouse cardiac endothelial cells (pEC) from our lab^6^. Briefly, GFP-positive cardiac endothelial cells from mT/mG-Cdh5Cre mice were isolated by fluorescence assisted cell sorting (FACS) after mechanical and enzymatic digestion of heart tissue. RNA processing, library preparation and sequencing were conducted as described above. RNAseq data are available at the NCBI Gene Expression Omnibus repository (BioProject ID PRJNA354710).

### Bioinformatics analysis and statistics

RNAseq data were analyzed using a Galaxy platform^28^. Adapters were trimmed from reads using Trim Galore and reads were mapped to the *Mus musculus* genome (mm9) using STAR^29^. After duplicate read removal transcript abundance was estimated as fragments per kilobase of transcript per million fragments mapped using Cufflinks^30^. Differential expression of Ensembl mm9 annotated genes^31^ was determined using DEseq. 2^32^ with q < 0.05 considered to be significant. RNA class assignment was obtained from the Ensembl mm9 annotation^31^. Enrichment of molecular pathways from Gene Ontology or Kyoto Encyclopedia of Genes and Genomes (KEGG) was analyzed using ClueGO^33^ or DAVID functional annotation tools^34^.

Unless otherwise stated *in vitro* experiments were performed in triplicate. Statistical analysis was performed using GraphPad Prism 5.04 with a p value < 0.05 was considered significant.

### Ethical approval

All animal procedures were approved by the responsible animal care committees (Regierungspraesidium Freiburg, Germany) and they conformed to the Guide for the Care and Use of Laboratory Animals published by the US National Institutes of Health (2011).

## Supplementary information

Supplementary Data.
