# Supplementary material for: Gene expression in immortalized versus primary isolated cardiac endothelial cells
Source: Sci Rep. 2020 Feb 10;10:2241. doi: 10.1038/s41598-020-59213-x (PMC7010830; doi:10.1038/s41598-020-59213-x)
Supplement: Supplementary file 1 — Supplementary Data. [file 41598_2020_59213_MOESM1_ESM.docx]

**Gene expression in immortalized versus primary isolated cardiac endothelial cells**

**SUPPLEMENTARY MATERIAL**

Lisa Deng^1^, Luisa Pollmeier^1^, Qian Zhou^2^, Stella Bergemann^1^, Christoph Bode^2^, Lutz Hein^1,3^, Achim Lother^1,2^

^1^ Institute of Experimental and Clinical Pharmacology and Toxicology, Faculty of Medicine, University of Freiburg, Germany

^2^ Heart Center Freiburg University, Department of Cardiology and Angiology I, Faculty of Medicine, University of Freiburg, Germany

^3^ BIOSS Centre for Biological Signaling Studies, University of Freiburg, Germany

**Supplementary Methods**

Total RNA was extracted from MCEC using RNeasy Mini Kit (Qiagen) according to manufacturer’s instruction and used for the synthesis of cDNA using the QuantiTect Reverse Transcription Kit (Qiagen). Quantitative real-time polymerase chain reaction was carried out using a CFX96 Touch Real-Time PCR Detection System (Bio-Rad) and the SsoAdvanced Universal SYBR Green Supermix (Bio-Rad). Gene expression analyses were normalized to expression of *Rps29* (Ribosomal Protein S29). Primer sequences are given in Supplementary Table 1.

**Supplementary Table 1: Primer sequences**

| **Gene** | **Forward primer** | **Reverse primer** |
| --- | --- | --- |
| *Rps29* | ATGGGTCACCAGCAGCTCTA | AGCCTATGTCCTTCGCGT ACT |
| *Cdh5* | TATAGGGACCTCTGTCATCCG TG | CGATTTGGTACAAGACAG TGGC |
| *Pecam1* | AACAGAGCCAGCAGTATGAG | ATGACCACTCCAATGACA ACCA |
| *Kdr* | TTTGGCAAATACAACCCTTCAGA | GCAGAAGATACTGTCACCACC |

**Supplementary Figure 1**

**
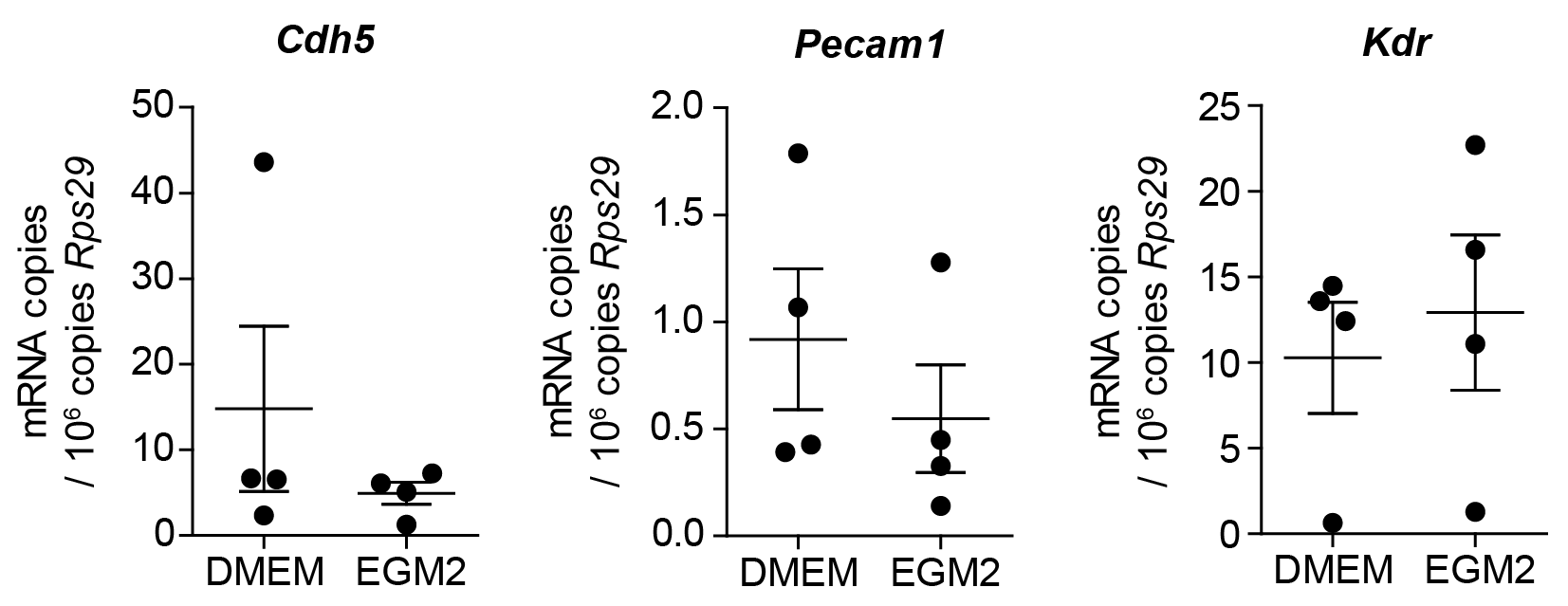
**

**Supplementary Figure 1: Culture medium-dependent expression of endothelial cell marker genes.** Immortalized mouse cardiac endothelial cells were cultured in either Dulbecco's Modified Eagle Medium with 5% FCS (DMEM) or in Microvascular Endothelial Cell Growth Medium-2 with 5% FCS and growth factor supplement (EGM2, Lonza) for 3 days. Expression of the typical endothelial cell marker genes cadherin 5 (Cdh5), platelet endothelial cell adhesion molecule 1 (Pecam1) or vascular endothelial cell growth factor receptor 2 (Kdr) was determined by qRT-PCR. Mean ± SEM. n = 4 per group. *Rps29*, Ribosomal protein S29.
